# Design and fabrication of a 3D–printed oral stent for head and neck radiotherapy from routine diagnostic imaging

**DOI:** 10.1186/s41205-017-0021-4

**Published:** 2017-11-16

**Authors:** Christopher T. Wilke, Mohamed Zaid, Caroline Chung, Clifton D. Fuller, Abdallah S. R. Mohamed, Heath Skinner, Jack Phan, G. Brandon Gunn, William H. Morrison, Adam S. Garden, Steven J. Frank, David I. Rosenthal, Mark S. Chambers, Eugene J. Koay

**Affiliations:** 1 Department of Radiation Oncology, University of Minnesota, Minneapolis, MN USA; 2 Department of Radiation Oncology, The University of Texas MD Anderson Cancer Center, 1220 Holcombe Blvd, MS97, Houston, TX 77030 USA; 3 Department of Head and Neck Surgery, Section of Oral Oncology, University of Texas MD Anderson Cancer Center, Houston, TX USA

**Keywords:** 3D printing, Oral stent, Head and neck cancer, Radiation

## Abstract

**Background:**

Oral stents have been shown to reduce the deleterious effects of head and neck radiotherapy through the displacement of normal tissues away from the areas of high dose irradiation. While these stents are commonly used in the treatment of patients with head and neck cancer at many large academic cancer centers, their use is much more limited outside of these institutions due to the time and expertise required for their fabrication.

**Results:**

In the study, we describe a novel method to design and manufacture oral stents from routine computed tomography (CT) imaging studies through the use of 3D printing technologies.

**Conclusion:**

Our proposed method may help to greatly expand access to these beneficial devices for patients undergoing radiation treatment at centers without access to dental and oral/maxillofacial specialists.

## Background

There are an estimated 50,000 new cases of head and neck cancer diagnosed in the United States each year and over 10 times that number of cases globally [1]. The majority of these patients will receive radiotherapy at some point in their treatment course as either definitive or adjuvant therapy. While radiotherapy is very effective in eradicating disease, one of the major dose-limiting factors is the tolerance of the adjacent uninvolved tissue [2–6]. Irradiation of the surrounding structures in the treatment of head and neck malignancies can give rise to numerous acute and late-term toxicities, including mucositis, dysgeusia, dysphagia, xerostomia, soft tissue necrosis and osteoradionecrosis. A relatively simple yet highly effective method to reduce radiation-induced toxicity is through the physical displacement of adjacent tissues away from the tumor using a customized oral stent [7–10].

Oral stents have been used for several decades in patients undergoing radiotherapy for head and neck malignancies. Traditionally, these devices are made by dentists with oncology-specific training who collaborate with the treating radiation oncologist. To fabricate an oral stent, the dentist obtains an impression of the patient’s teeth which is used to create a model of the appropriate mandible-maxillary relationship. The dentist then uses this model to hand-sculpt the stent with the desired incisal opening, tongue positioning and soft tissue displacement. The benefits of oral stents fabricated in this fashion is that they provide reliable and reproducible jaw positioning since they are derived from patient-specific geometry. The drawback of these devices is that they do require at least two separate appointments for the patient and are labor intensive and time consuming for the dentist to fabricate. Given this and the lack of experience constructing these devices among most general dentists, they are not routinely used in the community outside of the large high-volume academic center setting despite their demonstrated benefits in reducing treatment-related toxicity.

The objective of this study was to develop a computer-aided design (CAD) and 3D printing technology workflow to create customized oral stents for head and neck radiotherapy purposes.

## Methods

### Delineation of the dental anatomy

**Fig. 1:**
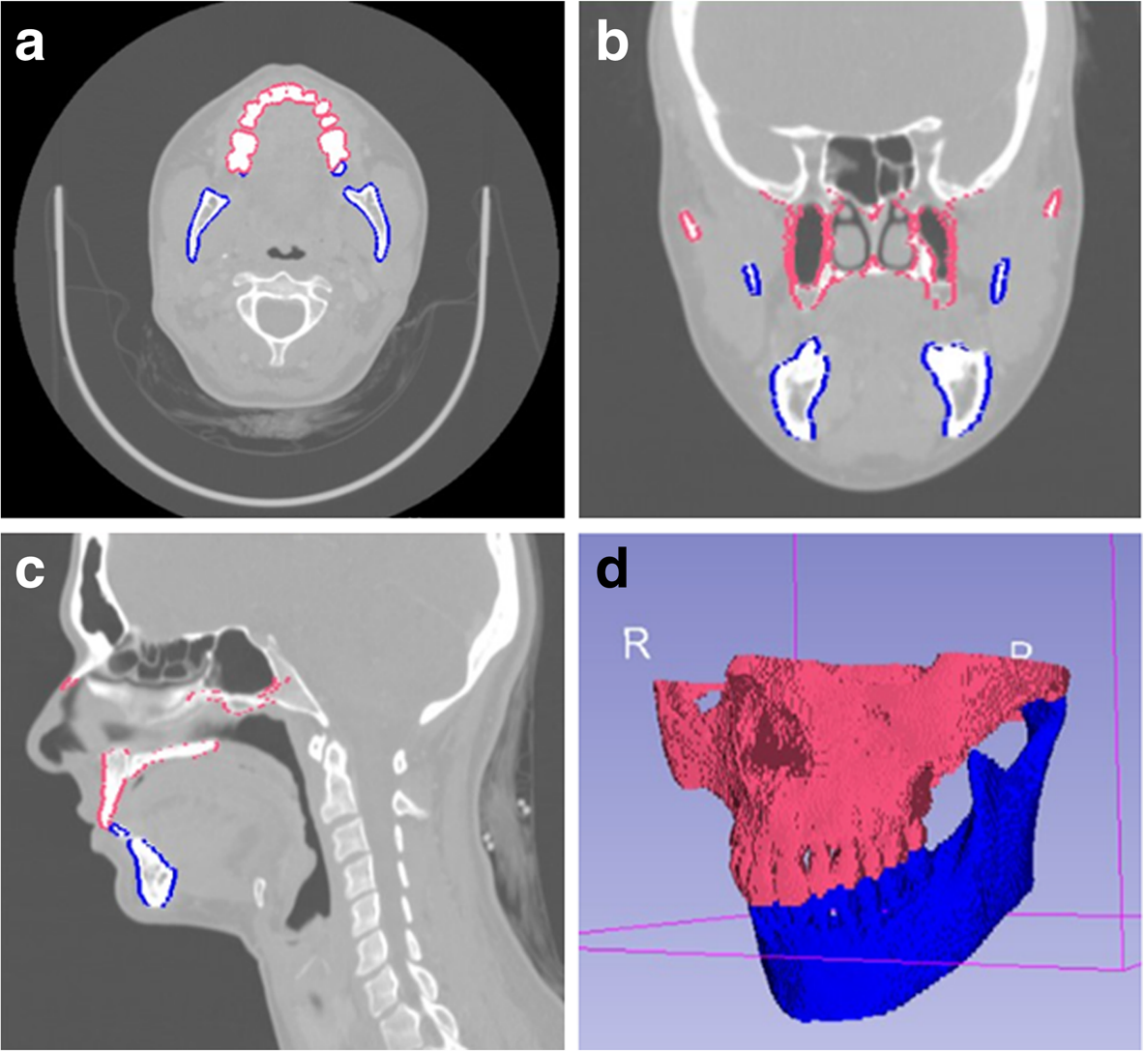
**a**) Axial, **b**) coronal and **c**) sagittal CT images obtained from 3D Slicer depicting the maxillary and mandibular anatomy with **d**) the corresponding 3D reconstruction

Routine diagnostic computed tomography (CT) images were obtained from a patient undergoing treatment of a primary head and neck malignancy under a protocol approved by the Institutional Review Board at MD Anderson Cancer Center. Selection of the study patient was made based on the absence of significant dental artifact and the availability of diagnostic imaging with adequate sampling (≤1 mm slice thickness) through the region of the maxillary and mandibular dentition. The maxilla and mandible were individually contoured and segmented as separate structures using the Velocity oncology imaging informatics system (Varian Medical Systems, Palo Alto, CA) and exported to the stereolithography (STL) file format using the 3D Slicer open source medical imaging software platform (Fig. 1) [11].

**Fig. 2:**
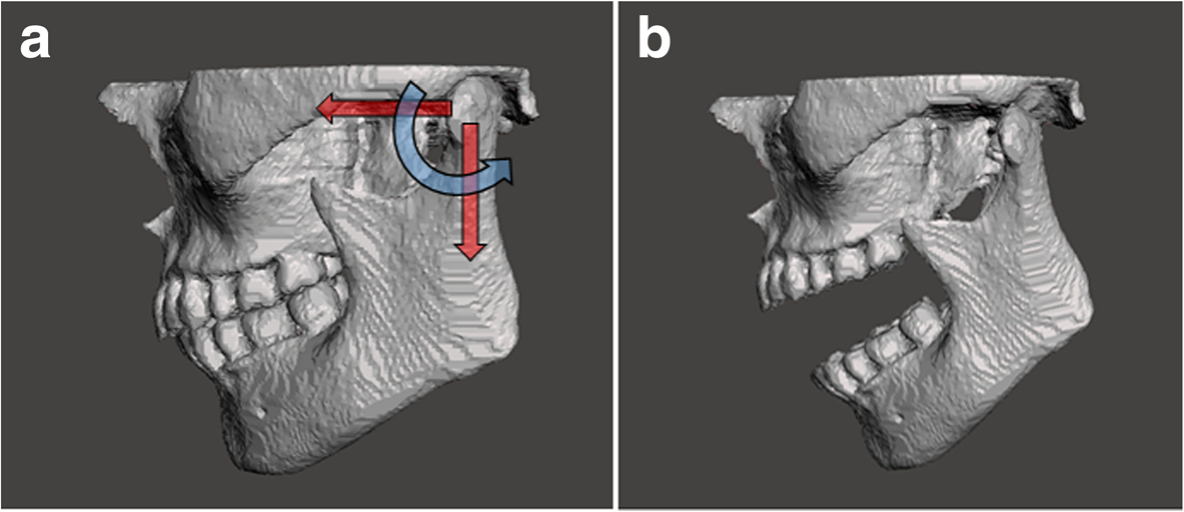
**a**) 3D CAD model obtained from the CT image dataset. The mandible was rotated and translated anteriorly and inferiorly to produce **b**) the jaw position with the desired incisal opening

The STL files containing the separate mandible and maxillary volumes were imported into 3D modeling software (Meshmixer, Autodesk Inc., San Rafael, CA). The mandible was treated as a rigid body and rotated and translated along the axis of the temporomandibular joint (TMJ) to simulate physiologic mandibular kinematics [12–14]. An incisal distance of 20 mm was used to approximate the desired jaw opening for radiotherapy treatment (Fig. 2).

### Oral stent design and fabrication

A mouth-opening, tongue-depressing stent was created in the same fashion as previously described by Kaanders et al. [8]. This type of stent consists of a plane that is in contact with the mandibular dentition and extends posteriorly beyond the level of the second molars to provide inferior displacement of the ventral surface of the tongue. On the lateral aspect of the stent, two struts in contact with the posterior maxillary dentition provide the desired degree of mouth-opening.

**Fig. 3:**
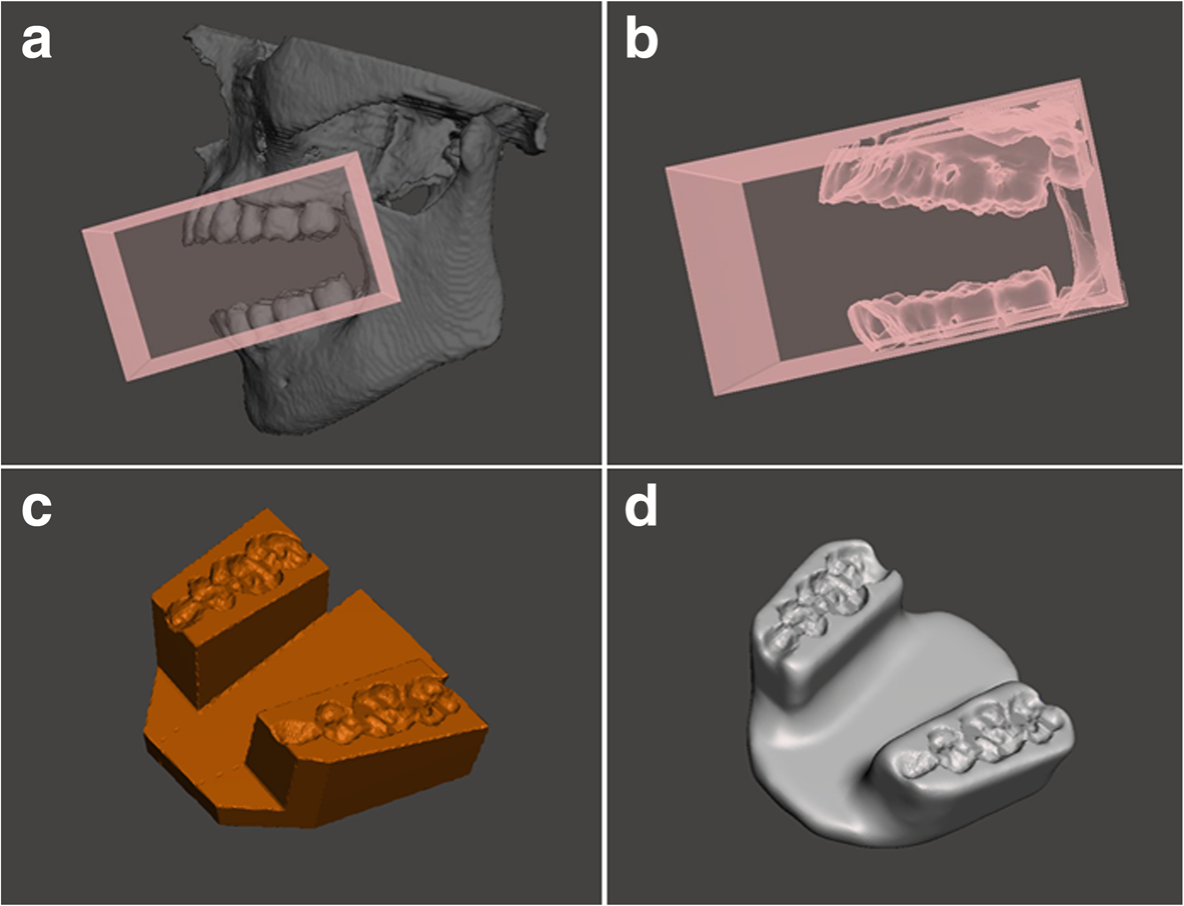
Graphical depiction of the process by which the stent is created from the 3D CAD maxillo-mandibular model. **a**) The rectangular template is overlaid with the mandibular and maxillary dentition to ensure coverage of the entire occlusal surfaces. **b**) The digital impression of the dentition with the selected incisal opening is created through Boolean subtraction of the patient’s dental anatomy from the template. **c**) Removal of the excess material to produce a mouth-opening, tongue-depressing stent. **d**) Smoothing of the external surfaces of the stent created in **c**) to produce the final product ready for 3D printing

To create a CAD model of the oral stent, a digital “impression” of the dentition was created from the maxillo-mandibular relationship described previously. A rectilinear volume template was overlaid with the dentition with visual verification to ensure all of the occlusal surfaces were within the selected region. Negative impressions of the patient’s dental anatomy with the desired mouth opening were obtained through Boolean subtraction of the patient’s dental anatomy from the rectilinear template. The excess material from the impression block was digitally removed to create the desired structure of the stent followed by post-processing steps to smooth the stent surfaces in contact with the oral mucosa. The major stages of the oral stent design process are depicted in Fig. 3.

## Results

**Fig. 4:**
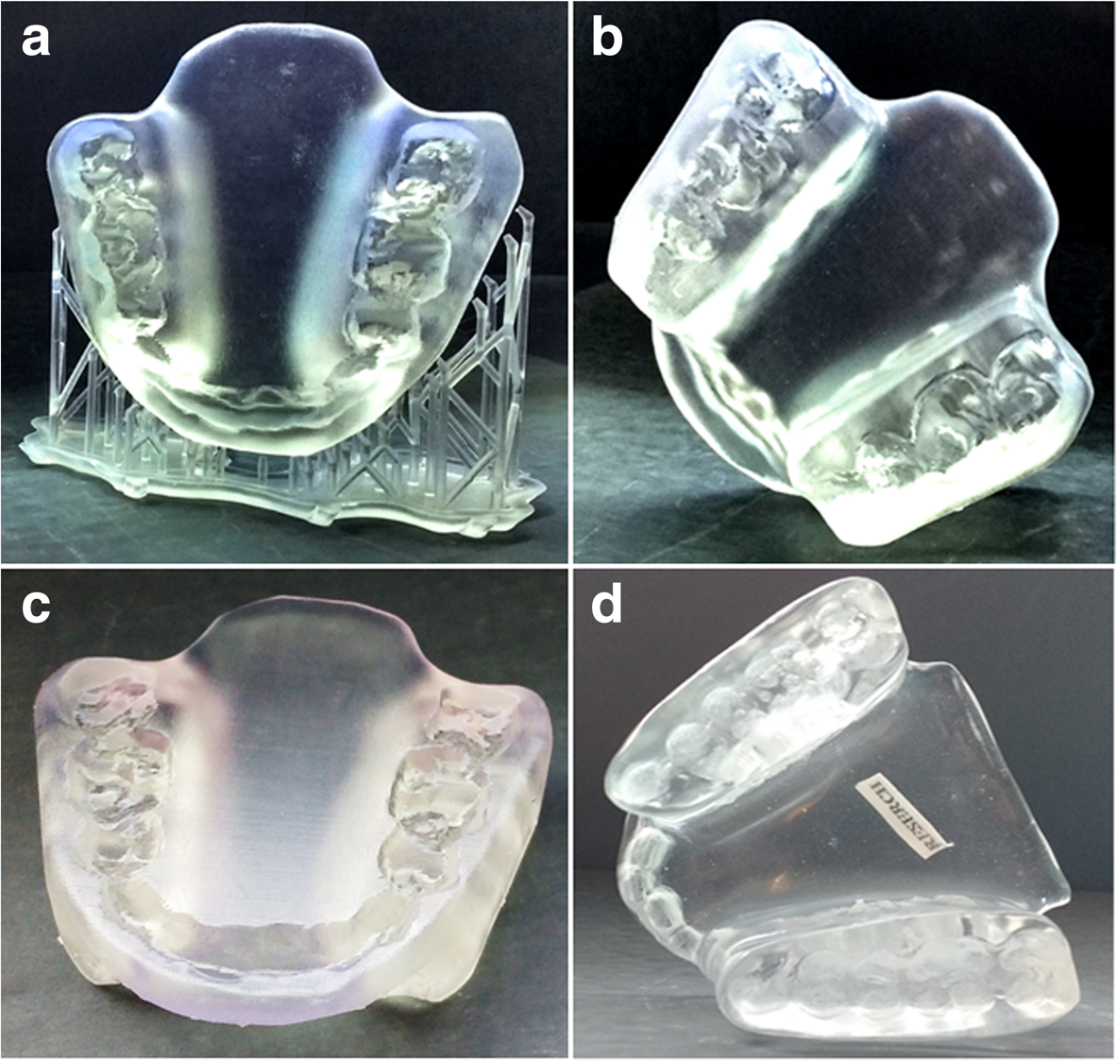
**a**) The 3D printed stent with support structures immediately following removal from the printer. The **b**) maxillary and **c**) mandibular occlusal surfaces following support structures removal and post-processing. **d** A reference oral stent as fabricated by dental oncology at MD Anderson Cancer Center

The CAD model of the oral stent was fabricated using the commercially-available Form 2 stereolithography printer (Formlabs Inc., Somerville, MA). Prior to printing, the STL file of the stent was uploaded to the PreForm software package (Formlabs) and manually positioned and oriented to minimize support structure attachment to the surfaces in contact with either the teeth or oral mucosa. This was performed both to minimize potential discomfort caused by local irregularities in contact with the oral mucosa as well as to prevent local geometric distortion of the stent near the support attachment sites which could reduce the fidelity of the stent-occlusal surface junction. The stent was printed using a Formlabs standard clear resin with a 50-μm layer thickness. After completion of printing, post-processing steps including removal of the support structures, washing and removal of residual uncured resin followed by sanding and polishing of the stent surfaces were performed to produce the final product shown in Fig. 4.

## Discussion

The predominant factor which limits the widespread utilization of oral stents for head and neck radiotherapy in the community setting is a lack of available dental or oral/maxillofacial professionals with knowledge and expertise in the creation of these devices. In this study, we have demonstrated the feasibility of utilizing 3D printing technology to create oral stents for use in patients undergoing head and neck radiotherapy using routine diagnostic CT imaging studies. Our technique is unique because it does not require the physical presence of the patient in order to fabricate the stent. Typically, oral stent fabrication requires at least 2 appointments with the patient to obtain dental impressions and assess the fit of the device. Our proposed method eliminates several of these steps and thus minimizes treatment delays.

There are several technical considerations associated with the design and fabrication of a 3D printed oral stent for use during head and neck radiotherapy treatment. Most importantly among these is the quality of the available diagnostic imaging which in turn directly affects the precision of the final oral stent product. We specifically selected a patient who had undergone diagnostic CT imaging with 1 mm slice thickness through the mandibular and maxillary dentition. While this procedure could be performed on imaging sets with greater slice thicknesses, the corresponding larger voxel size would limit the accuracy in defining the occlusal surfaces of the teeth. This in turn could produce a less robustly-fitting stent. Additionally, our selected patient had no significant imaging artifacts from the presence of dental hardware or amalgam. Similar to more sparely-sampled datasets described above, the presence of significant beam-hardening artifact on CT imaging may reduce the accuracy of the delineation of the mandibular and maxillary dentition. While it is not uncommon for many patients with head and neck cancer to have some degree of dental artifact, advances in software post-processing and use of dual-energy diagnostic CT imaging may help to reduce the impact of these artifacts on delineation and reconstruction of the relevant dental anatomy [15]. For patients who remain unsuitable for stent creation from diagnostic imaging, we are currently exploring additional methods, including optical imaging techniques, to incorporate into our current workflow.

A second consideration is the minimization or prevention of propagated errors from the initial segmentation to modeling and printing of the final product. Currently, there is no single software platform which offers streamlined end-to-end capabilities for creation of an oral stent from diagnostic imaging data. We instead utilized a mixture of robust proprietary and open-source/free software to segment the dental anatomy, convert the resultant DICOM data to an STL format and design the stent. To prevent errors introduced in the delineation of the dental anatomy, all segmentation was performed and verified by an experienced radiation oncologist before conversion to an STL mesh. Design of the oral stent was performed using Autodesk Meshmixer which is a free albeit powerful 3D modeling and sculpting software that has been used to make high-fidelity patient-specific coronary vasculature and dental models [16, 17]. Printing of the final device was performed using the Form2 desktop SLA printer which is approved for dental applications with 3D printed objects demonstrating dimensional accuracy within 50–100 μm.

Another important technical consideration is the choice of materials used in the fabrication of the 3D printed oral stent. The selected materials and printing methods must produce a stent which is sufficiently rigid to resist deforming under physiologic loading. Such deformation in stent geometry would result in unstable day-to-day jaw positioning which in turn could potentially adversely affect the quality of the delivered radiation treatment. The stent must also be fabricated from a biologically inert, non-toxic material to minimize potential harm to the patient. Fortunately, there are several commercially-available materials specifically designed and approved for dental applications which satisfy the aforementioned criteria. Although we used a standard Formlabs clear resin in the fabrication of the stent described in this manuscript, we have recently begun printing oral stents with an approved dental resin for the Form2 platform using an identical workflow.

The last technical consideration is the time required for creation of the device. Treatment delays in the initiation of radiotherapy have been shown to adversely impact survival for patients with head and neck malignancies [18, 19]. It is therefore imperative that any innovative process be capable of fabrication of these devices in a timely manner. Using the technique discussed in this manuscript, we were able to produce the 3D printed stent depicted in Fig. 4, from the initial image segmentation to the completion of the post-processing steps, in less than eight hours. We anticipate that this required time will dramatically shrink with additional optimization and automation of our current workflow and allow these devices to be created in an on-demand fashion for expedited initiation of oncologic-directed therapies.

## Conclusion

In summary, we have created a novel method by which to create 3D printed oral stents for use in patients receiving head and neck radiotherapy. We are currently in the process of prospectively evaluating the fit and comfort of our 3D printed stents as compared with the traditional dental-fabricated devices in a cohort of patient receiving radiotherapy at MD Anderson Cancer Center and will report these results separately. We believe that, by producing an inexpensive and easily fabricated customized oral stent for head and neck radiotherapy, we will dramatically lower the barrier to widespread adoption of these useful devices in the community setting.

## Abbreviations

CAD: Computer aided design
CT: Computed tomography
STL: Stereolithography
